# Next-Generation Immune Checkpoint Inhibitors in Gastrointestinal Cancers: Mechanisms, Resistance, and Emerging Therapeutic Strategies

**DOI:** 10.3390/cancers18162593

**Published:** 2026-08-12

**Authors:** Mariam Ismail, Zaid Alabed, Khaled Alhallaq, Ebtesam Al-Najjar, Nour Mustafa, Yacoub Aldroubi, Yazan Hamdaneh, Abdullah Esmail

**Affiliations:** 1 Faculty of Medicine, Delta University for Science and Technology, Gamasa 11152, Egypt; 2 Faculty of Medicine, The University of Jordan, Amman 11942, Jordan; 3 Department of Medicine, Houston Methodist Neal Cancer Center, Houston Methodist Hospital, Houston, TX 77030, USA; 4 Department of Medicine, Methodist Healthcare System of San Antonio, TX 78229, USA; 5 Department of Medicine, Weill Cornell Medical College, New York, NY 10065, USA

**Keywords:** ICIs, GI oncology, TIGIT, ctDNA, immune checkpoint inhibitors

## Abstract

Immune checkpoint inhibitors (ICIs) have significantly changed the treatment landscape of several gastrointestinal (GI) malignancies, particularly microsatellite instability-high (MSI-H) and mismatch repair-deficient (dMMR) tumors. However, most GI cancers remain resistant to current PD-1/PD-L1-based immunotherapy because of complex and highly suppressive tumor microenvironments characterized by immune stromal exclusion, myeloid-driven immune suppression, defective antigen presentation, and adaptive immune resistance mechanisms. Emerging evidence suggests that alternative inhibitory pathways, including lymphocyte activation gene-3 (LAG-3), T-cell immunoreceptor with immunoglobulin and ITIM domain (TIGIT), T-cell immunoglobulin and mucin-domain containing-3 (TIM-3), and V-domain Ig suppressor of T-cell activation (VISTA), contribute substantially to persistent T-cell dysfunction and resistance to checkpoint blockade. This review summarizes the immune landscape of GI malignancies and discusses the biological mechanisms underlying resistance to current ICIs. We highlight the evolving role of next-generation immune checkpoints, ongoing clinical development of novel inhibitors, and emerging combination strategies involving chemotherapy, anti-angiogenic therapy, radiation, bispecific antibodies, and tumor microenvironment modulation. In addition, we discuss current limitations in biomarker development and the growing role of circulating tumor DNA, spatial immune profiling, and multi-omics approaches in patient selection. Finally, we explore future directions aimed at improving precision immunotherapy and expanding durable responses across GI cancers.

## Simple Summary

Gastrointestinal cancers remain among the most challenging diseases to treat because many tumors contain complex immune system suppression mechanisms that stop the body from destroying cancer cells. While current immunotherapy drugs have transformed care for a small group of patients, most gastrointestinal tumors quickly resist these treatments. This review explains how cancer cells bypass standard therapies and highlights next-generation immune targets currently being developed to overcome this resistance. We examine how combining new immune-boosting drugs with traditional treatments like chemotherapy, radiation, and novel targeted therapies can weaken cancer defenses. Finally, we explore advanced blood tests and digital tissue profiling methods designed to help doctors predict which patients will benefit most from these therapies. By connecting basic tumor biology with innovative clinical strategies, this review aims to help researchers design smarter, more focused and effective treatment combinations for patients with gastrointestinal cancers.

## 1. Introduction

Gastrointestinal (GI) malignancies remain a leading cause of cancer-related morbidity and mortality worldwide despite major advances in systemic therapy and perioperative care [1,2]. Immune checkpoint inhibitors (ICIs) have transformed cancer treatment by restoring antitumor T-cell activity and producing durable responses in selected malignancies [3]. In GI oncology, however, the benefit of current ICIs remains largely limited to specific molecular subgroups, particularly microsatellite instability-high (MSI-H) and mismatch repair-deficient (dMMR) tumors [4,5]. Most GI cancers, including microsatellite-stable (MSS) colorectal cancer and pancreatic ductal adenocarcinoma, demonstrate either primary or acquired resistance to PD-1/PD-L1 blockade because of poor T-cell infiltration, stromal exclusion, defective antigen presentation, and highly suppressive tumor microenvironments [6].

Increasing evidence suggests that resistance to checkpoint inhibition is driven by multiple overlapping mechanisms involving T-cell exhaustion, adaptive immune escape, myeloid-mediated immune suppression, and compensatory activation of alternative inhibitory pathways [3,7]. Exhausted tumor-infiltrating lymphocytes frequently co-express inhibitory receptors such as lymphocyte activation gene-3 (LAG-3), T-cell immunoreceptor with immunoglobulin and ITIM domain (TIGIT), T-cell immunoglobulin and mucin-domain containing-3 (TIM-3), and V-domain Ig suppressor of T-cell activation (VISTA), all of which contribute to persistent immune dysfunction within the tumor microenvironment [8].

As a result, growing attention has shifted toward next-generation checkpoint inhibitors and rational combination strategies aimed at overcoming immune resistance in GI cancers. Approaches involving dual checkpoint blockade, chemotherapy, anti-angiogenic therapy, radiation, bispecific antibodies, and tumor microenvironment modulation are currently under active investigation [9,10].

This review summarizes the immune landscape of GI malignancies, mechanisms of resistance to current ICIs, emerging next-generation checkpoint targets, evolving combination immunotherapy strategies, and current challenges in biomarker development and patient selection. This review also hopes to expand on past research by focusing on combination strategies specifically and new integrative frameworks [11].

## 2. Immune Landscape of Gastrointestinal Cancers

The limited efficacy of immune checkpoint inhibitors (ICIs) in many GI malignancies reflects the highly suppressive nature of the gastrointestinal tumor immune microenvironment (TIME) [12,13,14,15,16,17,18]. Unlike highly immunogenic tumors such as melanoma, many GI cancers exhibit poor cytotoxic T-cell infiltration, dense stromal remodeling, myeloid-driven immune suppression, and adaptive resistance mechanisms that promote immunologically “cold” phenotypes [19,20]. Consequently, meaningful responses to checkpoint blockade are largely confined to a subset of patients, particularly those with microsatellite instability-high (MSI-H) or mismatch repair-deficient (dMMR) tumors [19,21].

The GI tumor microenvironment contains multiple immunosuppressive cell populations, including regulatory T cells (Tregs), tumor-associated macrophages (TAMs), myeloid-derived suppressor cells (MDSCs), cancer-associated fibroblasts (CAFs), and regulatory dendritic cells. Together with abnormal cytokine signaling, hypoxia, and metabolic stress, these components impair antigen presentation, suppress cytotoxic T-cell activity, and promote T-cell exhaustion [4,6]. In parallel, stromal and tumor-intrinsic signaling pathways such as TGF-β, PI3K/AKT/mTOR, and MAPK/ERK contribute to fibrosis, immune exclusion, and resistance to immunotherapy [22,23]. Defective antigen presentation, impaired interferon signaling, epigenetic reprogramming, and compensatory checkpoint upregulation further reduce responsiveness to PD-1/PD-L1 blockade [19,24].

Understanding these mechanisms has become central to the development of next-generation immunotherapeutic strategies aimed at restoring antitumor immunity and remodeling the tumor microenvironment through stromal targeting, myeloid reprogramming, anti-angiogenic therapy, epigenetic modulation, and multi-checkpoint blockade [4,6].

### 2.1. T-Cell Activation and Immune Surveillance

The immune system plays a central role in recognizing and eliminating malignant cells through immune surveillance, a concept later expanded into cancer immunoediting, which describes tumor elimination, equilibrium, and escape phases [25,26]. Experimental studies demonstrated that immunodeficient mice develop higher rates of spontaneous and carcinogen-induced tumors, while adaptive immunity can maintain occult malignancies in equilibrium states [27,28]. In humans, tumor-infiltrating lymphocytes have been associated with improved outcomes across multiple cancers, supporting the importance of antitumor immunity in cancer control [29].

Cytotoxic CD8+ T cells are the primary mediators of antitumor immunity and recognize tumor-associated antigens presented on major histocompatibility complex class I (MHC-I) molecules [30,31]. Effective activation requires antigen recognition through the T-cell receptor together with co-stimulatory signaling through CD28 and B7 ligands [32,33]. Activated T cells subsequently infiltrate tumors and induce tumor-cell death through perforin- and granzyme-mediated cytotoxicity while secreting interferon-γ (IFN-γ), which enhances antigen presentation and immune cell recruitment [34,35,36,37,38].

Under physiological conditions, immune checkpoints regulate T-cell activation to maintain self-tolerance and prevent excessive immune responses [39]. CTLA-4 and PD-1 are key inhibitory receptors that suppress T-cell activation and effector activity [40,41]. However, tumor cells frequently exploit these pathways through PD-L1 upregulation driven by oncogenic signaling, inflammatory cytokines, and epigenetic regulation, thereby promoting immune escape and resistance to antitumor immunity [42,43].

### 2.2. Immune Checkpoints and Immune Homeostasis

Immune checkpoints are inhibitory receptors that regulate T-cell activation and maintain immune homeostasis [44,45]. Tumor cells frequently exploit these pathways to suppress antitumor immunity and evade immune surveillance [46,47].

Among the most well-characterized checkpoints are programmed death-1 (PD-1) and cytotoxic T-lymphocyte-associated protein 4 (CTLA-4). PD-1 signaling suppresses T-cell proliferation, cytokine production, and cytotoxic function following interaction with PD-L1 or PD-L2 on tumor cells and antigen-presenting cells [46,47]. In contrast, CTLA-4 primarily regulates early T-cell priming by competing with CD28 for B7 ligand binding [48].

Therapeutic blockades of PD-1, PD-L1, and CTLA-4 has transformed cancer treatment and produced durable responses across multiple malignancies. Agents including nivolumab, pembrolizumab, atezolizumab, durvalumab, and ipilimumab have demonstrated meaningful survival benefits in selected cancers [49]. In GI oncology, the greatest efficacy has been observed in MSI-H/dMMR tumors [50,51]. However, most GI malignancies remain poorly responsive to checkpoint blockade, highlighting the complexity of immune resistance and the need for improved biomarker-driven and combinational strategies [52,53].

### 2.3. T-Cell Exhaustion

Chronic antigen exposure within the tumor microenvironment drives T cells into a dysfunctional state known as T-cell exhaustion, which is characterized by impaired proliferation, reduced cytokine production, diminished cytotoxic activity, and persistent expression of inhibitory receptors including PD-1, LAG-3, TIM-3, and TIGIT [54,55,56]. Co-expression of these checkpoints reflects activation of complementary suppressive pathways that reinforce immune dysfunction [8].

Exhausted CD8+ T cells exist along a heterogeneous differentiation spectrum. Progenitor-like exhausted T cells retaining TCF-1 expression remain partially responsive to PD-1 blockade, whereas terminally exhausted T cells exhibit profound dysfunction and limited reversibility [57,58]. This exhausted state is further stabilized by epigenetic reprogramming and loss of CD28 expression, both of which have been associated with resistance to PD-1 blockade [59,60,61].

These findings have become central to the development of next-generation immunotherapeutic strategies. Dual checkpoint blockade targeting PD-1 together with LAG-3, TIGIT, TIM-3, or VISTA aims to overcome interconnected inhibitory pathways sustaining T-cell exhaustion [8]. Combined PD-1 and LAG-3 inhibition demonstrated synergistic restoration of antitumor immunity and led to the first FDA-approved next-generation checkpoint combination with nivolumab plus relatlimab [62]. Additional approaches, including epigenetic modulation, cytokine-based therapies, and engineered cellular therapies, are also under investigation to restore exhausted T-cell function and improve immunotherapy responsiveness [63,64,65].

### 2.4. Tumor Microenvironment in GI Malignancies

The TME of GI cancers is highly immunosuppressive, limiting antitumor immunity and ICI responsiveness. As detailed in Table 1, key suppressive elements include Tregs, MDSCs, TAMs, CAFs, and immunosuppressive cytokines that impair antigen presentation, promote T-cell exhaustion, and create physical barriers to immune infiltration [66,67].

PDAC exemplifies the “cold” phenotype: extensive desmoplastic stroma, myeloid-dominant infiltrates, and CXCL12-mediated T-cell sequestration [96,97,98,99]. In contrast, MSI-H/dMMR CRCs display an inflamed, checkpoint-restrained microenvironment with high CD8+ T-cell infiltration and upregulation of PD-1, TIM-3, and CTLA-4 [64,73,87]. These features underlie the improved responsiveness of MSI-H CRCs to checkpoint blockade, as demonstrated in the KEYNOTE-177 trial [95,100]. Together, these findings highlight the central role of the TME in shaping immunotherapy outcomes across GI malignancies.

### 2.5. Mechanisms of Immune Escape and Resistance

Resistance to immunotherapy in GI malignancies may be primary or acquired and arises through coordinated tumor-intrinsic and tumor-extrinsic mechanisms that impair effective antitumor immunity [3,101]. Tumor-intrinsic resistance mechanisms include defective antigen presentation, impaired interferon-γ (IFN-γ) signaling, and reduced tumor immunogenicity. Alterations involving β2-microglobulin (B2M), HLA class I molecules, and antigen-processing machinery impair presentation of tumor-associated antigens and have been linked to resistance to checkpoint blockade, particularly in MSI-H colorectal cancers under strong immune selective pressure [102,103,104]. Similarly, defects in IFN-γ/JAK/STAT signaling reduce MHC expression, impair T-cell recruitment, and decrease sensitivity to immune-mediated killing [105].

**Figure 1:**
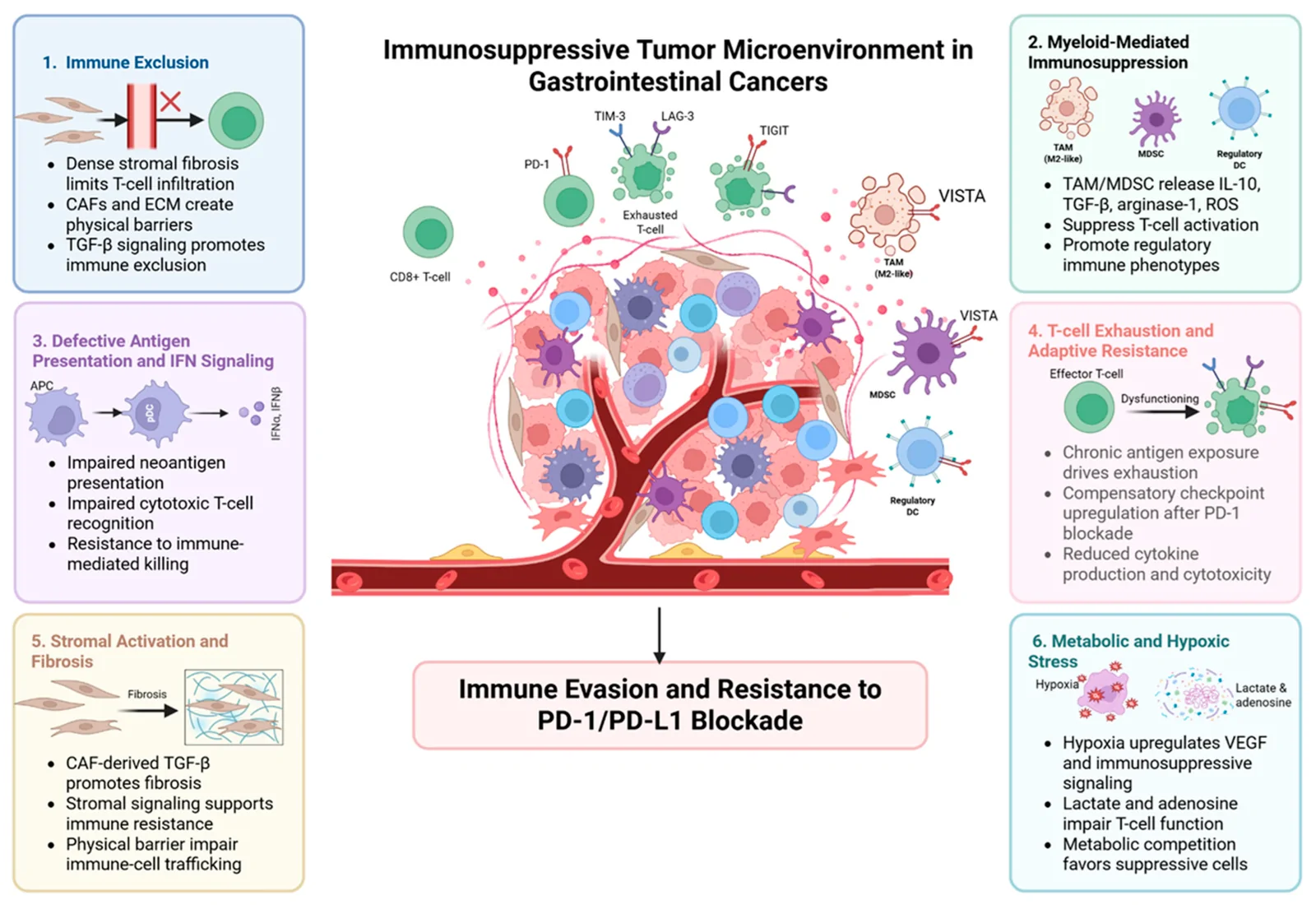
Mechanisms of immune resistance within the gastrointestinal tumor microenvironment [107].

At the same time, GI tumors establish highly suppressive tumor microenvironments, as seen in Figure 1, that promote immune exclusion and sustain T-cell dysfunction. MDSCs, Tregs, TAMs, and CAFs suppress antitumor immunity through cytokine secretion, metabolic competition, stromal remodeling, and impaired dendritic-cell function [4,106]. Additional processes including epithelial–mesenchymal transition, hypoxia, angiogenic signaling, and TGF-β activation further contribute to resistance to immune checkpoint blockade [86].

Adaptive resistance following PD-1 blockade has also emerged as a major mechanism of treatment failure. Exhausted tumor-infiltrating lymphocytes frequently upregulate alternative inhibitory checkpoints such as LAG-3, TIM-3, TIGIT, and VISTA, which cooperatively maintain immune dysfunction despite PD-1 inhibition [62,108]. Unlike PD-1 and CTLA-4, VISTA predominantly regulates myeloid-mediated immune suppression and has been associated with immunologically “cold” tumor microenvironments and resistance to checkpoint inhibition [109,110].

Collectively, these mechanisms help explain why durable responses to single-agent PD-1/PD-L1 blockade remains limited in many GI malignancies and provide the rationale for next-generation checkpoint inhibitors and rational combination immunotherapy strategies.

## 3. Limitations of Current Immune Checkpoint Inhibitors in GI Oncology

### 3.1. Current Role of PD-1/PD-L1 Blockade in GI Cancers

Immune checkpoint inhibitors targeting PD-1 and PD-L1 have significantly changed the treatment landscape of several GI malignancies and established new standards of care in selected settings. In advanced gastric and esophageal cancers, combination regimens incorporating pembrolizumab or nivolumab with chemotherapy have demonstrated meaningful survival benefits, particularly in tumors with elevated PD-L1 expression [111,112]. In hepatocellular carcinoma (HCC), combinations such as atezolizumab plus bevacizumab and nivolumab plus ipilimumab have further expanded the role of immunotherapy in advanced disease [15,113,114].

The greatest benefit from checkpoint inhibition in GI oncology has been observed in microsatellite instability-high (MSI-H) or mismatch repair-deficient (dMMR) colorectal cancers. In the KEYNOTE-177 trial, pembrolizumab significantly improved progression-free survival compared with chemotherapy in metastatic MSI-H/dMMR colorectal cancer, establishing PD-1 blockade as a first-line standard in this subgroup [93,115]. Similarly, nivolumab plus ipilimumab demonstrated durable clinical benefit and high response rates in MSI-H metastatic colorectal cancer [50].

Despite these advances, the efficacy of current checkpoint inhibitors in GI cancers remains highly variable. Durable responses are largely restricted to biomarker-defined subgroups, particularly MSI-H/dMMR and PD-L1–high tumors, while most GI malignancies derive only limited benefit from PD-1/PD-L1 blockade [19]. Microsatellite-stable (MSS) colorectal cancer, which represents the majority of metastatic colorectal cancers, remains largely resistant to checkpoint inhibition, with response rates below 10% [116,117]. Pancreatic ductal adenocarcinoma similarly demonstrates profound resistance due to dense stromal desmoplasia, low tumor immunogenicity, and highly suppressive tumor microenvironments [118].

Current biomarkers also remain imperfect predictors of response. Although MSI status and PD-L1 expression are clinically useful, substantial heterogeneity exists even within biomarker-defined groups, and tumor mutational burden has shown inconsistent predictive value in many MSS GI tumors [119,120]. Together, these limitations highlight the need for next-generation checkpoint inhibitors, rational combination strategies, and more refined biomarker-driven approaches to expand the benefit of immunotherapy across GI malignancies.

### 3.2. MSI-H Versus MSS Colorectal Cancer

The contrast between MSI-H and MSS colorectal cancer (CRC) exemplifies biomarker-driven immunotherapy responsiveness. As shown in Table 1, MSI-H CRCs display high TMB, abundant neoantigens, and dense CD8+ T-cell infiltration with elevated checkpoint expression [81], while MSS CRCs are characterized by T-cell exclusion and suppressive myeloid cells [4]. These immunologic differences translate into clinical benefit: in the KEYNOTE-177 trial, pembrolizumab significantly improved PFS versus chemotherapy in metastatic MSI-H/dMMR CRC [93,115], and nivolumab plus ipilimumab (CheckMate 8HW) demonstrated high response rates and increased progression-free survival, respectively [50]. By contrast, MSS CRC remains largely resistant to ICIs, with response rates below 10% [116,117]. Emerging strategies to overcome resistance in MSS CRC include combination immunotherapy, WNT/β-catenin inhibition, myeloid reprogramming, and stromal modulation [121,122].

### 3.3. Cold Tumors and Immune Exclusion

Many GI malignancies exhibit immune-excluded or immune-desert phenotypes, commonly referred to as “cold” tumors, which are characterized by limited cytotoxic T-cell infiltration and poor responsiveness to immune checkpoint inhibitors [67,123]. In immune-excluded tumors, T cells remain confined to the surrounding stroma and fail to penetrate tumor-cell nests, whereas immune-desert tumors show minimal T-cell infiltration throughout the tumor microenvironment [124]. These phenotypes are particularly common in pancreatic ductal adenocarcinoma and microsatellite-stable colorectal cancer, where immune exclusion contributes substantially to immunotherapy resistance [97,125].

Several mechanisms cooperate to maintain these cold immune states. Cancer-associated fibroblasts, extracellular matrix remodeling, and abnormal tumor vasculature create physical barriers that restrict immune cell trafficking into tumor tissue [126,127]. At the same time, TGF-β signaling suppresses cytotoxic T-cell recruitment and promotes immunosuppressive stromal and macrophage programs [128,129]. Immunosuppressive myeloid cells, regulatory T cells, and dysfunctional dendritic cells further impair antitumor immunity through cytokine secretion, metabolic suppression, and reduced antigen presentation [4,130].

Tumor-intrinsic pathways also contribute to immune exclusion. Activation of Wnt/β-catenin signaling, MAPK pathway alterations, PTEN loss, and epithelial–mesenchymal transition programs can impair dendritic-cell recruitment, suppress chemokine production, and reduce T-cell priming within the tumor microenvironment [131,132]. Together, these stromal, immune, and tumor-intrinsic mechanisms create a microenvironment that is resistant to single-agent checkpoint blockade and provide the rationale for combination strategies aimed at restoring T-cell infiltration and converting cold GI tumors into more immune-responsive phenotypes.

### 3.4. Adaptive Resistance Pathways

Adaptive resistance is a major limitation of immune checkpoint blockade, whereby tumors exposed to PD-1/PD-L1 inhibition develop compensatory mechanisms that sustain immune evasion despite therapy. One of the best-characterized mechanisms is the upregulation of alternative inhibitory checkpoints on tumor-infiltrating lymphocytes, including TIM-3, LAG-3, TIGIT, and VISTA [108,133]. Koyama et al. demonstrated that tumors progressing after anti-PD-1 therapy showed increased TIM-3 expression on T cells and that subsequent TIM-3 blockade improved survival in preclinical models, providing early evidence that checkpoint switching can mediate acquired resistance [108].

This compensatory biology provides the rationale for dual and multi-checkpoint blockade strategies. LAG-3 suppresses activated T-cell expansion and function through mechanisms distinct from PD-1, and combined PD-1/LAG-3 inhibition has demonstrated enhanced antitumor activity in both preclinical and clinical studies [62,134]. In gastroesophageal cancer, neoadjuvant nivolumab plus relatlimab showed encouraging pathological responses, supporting the potential role of LAG-3 targeting in GI malignancies [135]. However, the limited efficacy of nivolumab plus relatlimab in mismatch repair-proficient colorectal cancer highlights the ongoing challenge of overcoming resistance in immunologically cold tumors [136].

TIGIT and VISTA represent additional adaptive resistance pathways with distinct immunologic roles. TIGIT suppresses T-cell and natural killer cell activity through competition with the co-stimulatory receptor CD226 for CD155 binding and is frequently co-expressed with PD-1 on exhausted T cells [137,138]. In contrast, VISTA is predominantly expressed on myeloid cells and contributes to myeloid-mediated immune suppression and maintenance of cold tumor microenvironments. In gastric cancer, elevated VISTA expression has been associated with exhausted CD8+ T cells, regulatory T cells, M2 macrophages, and poorer outcomes during checkpoint inhibitor therapy [109,139].

Adaptive resistance may also arise through tumor-intrinsic alterations that develop under immune pressure, including defects in antigen presentation and IFN-γ/JAK/STAT signaling, both of which reduce sensitivity to checkpoint blockade [86,140]. In parallel, chronic antigen stimulation promotes stable epigenetic programs associated with terminal T-cell exhaustion that are only partially reversible with PD-1 inhibition [65,141]. Together, these mechanisms support the need for combination strategies targeting multiple resistance pathways, including alternative checkpoints, tumor-intrinsic immune escape, myeloid suppression, and T-cell epigenetic dysfunction.

### 3.5. Biomarker Limitations

Although PD-L1 expression, MSI/dMMR status, and tumor mutational burden (TMB) are clinically useful biomarkers for selecting patients for immune checkpoint inhibitors, each has important limitations when used alone in GI malignancies [120,142]. PD-L1 expression is widely used in gastroesophageal cancers, but its prognostic and predictive value varies depending on scoring methods, assay platforms, histologic subtype, and cutoff thresholds. While a higher PD-L1 combined positive score (CPS) is generally associated with greater benefit from PD-1/PD-L1 blockade, responses can still occur in PD-L1-low or negative tumors, limiting its reliability as a standalone biomarker [76,143]. In addition, PD-L1 expression shows substantial spatial and temporal heterogeneity and may differ between primary and metastatic lesions or change after systemic therapy [144].

MSI-H/dMMR status remains the strongest predictive and prognostic biomarker for checkpoint inhibitor responsiveness in GI oncology, particularly in colorectal cancer. However, MSI-H/dMMR tumors account for only a small proportion of metastatic GI cancers, and not all patients within this subgroup respond to immunotherapy [100,145]. Discordance between mismatch repair immunohistochemistry, PCR-based MSI testing, and next-generation sequencing can also lead to biomarker misclassification in selected cases [146,147].

TMB has emerged as a tissue-agnostic biomarker for immunotherapy response, but its role in GI cancers remains inconsistent. Although high TMB may correlate with increased neoantigen burden and improved response in some tumors, not all mutations produce immunogenic peptides capable of effective antigen presentation and T-cell recognition [148,149]. In addition, TMB thresholds vary across tumor types and sequencing platforms, and high TMB in microsatellite-stable GI cancers does not consistently predict benefit from checkpoint blockade [119,150].

These limitations highlight that no single biomarker fully reflects the complexity of antitumor immunity. As a result, current research is increasingly focused on composite and dynamic biomarkers, including immune gene-expression signatures, T-cell-inflamed profiles, spatial immune architecture, circulating tumor DNA kinetics, microbiome composition, and multi-omics models integrating tumor genomics with features of the immune microenvironment [24,151,152]. Together, these approaches may improve patient selection and help guide more personalized immunotherapy strategies in GI cancers.

## 4. Next-Generation Immune Checkpoints

### 4.1. LAG-3

#### 4.1.1. Biology and Mechanisms

Lymphocyte activation gene-3 (LAG-3) is an inhibitory immune checkpoint receptor expressed on activated T cells, regulatory T cells, natural killer cells, and dendritic cells [134,153]. Structurally related to CD4, LAG-3 binds major histocompatibility complex class II (MHC-II) molecules and suppresses T-cell activation and effector function [154]. Additional ligands, including fibrinogen-like protein 1 (FGL1), Galectin-3, and LSECtin, further enhance immune suppression within the tumor microenvironment [155,156]. Among these, FGL1 has been particularly associated with resistance to anti-PD-1 therapy and poor clinical outcomes [156].

#### 4.1.2. LAG-3 and T-Cell Exhaustion

LAG-3 is strongly linked to T-cell exhaustion and is commonly co-expressed with PD-1 and TIM-3 on dysfunctional tumor-infiltrating lymphocytes [62]. Chronic antigen exposure within the tumor microenvironment drives progressive upregulation of LAG-3, leading to impaired cytotoxic activity and persistent immune dysfunction. Preclinical studies have shown that combined PD-1 and LAG-3 blockade produces stronger antitumor responses than inhibition of either pathway alone, supporting the rationale for dual checkpoint blockade strategies [62,157].

#### 4.1.3. Clinical Development

Relatlimab, a monoclonal antibody targeting LAG-3, became the first clinically successful next-generation checkpoint inhibitor after the RELATIVITY-047 trial demonstrated improved progression-free survival with nivolumab plus relatlimab compared with nivolumab alone in advanced melanoma [158]. LAG-3-targeted therapies are now being explored in several GI malignancies. This translation has been challenging, however, as even though in early gastroesophageal cancer, neoadjuvant nivolumab plus relatlimab showed encouraging pathological responses, phase II trials like RELATIVITY-060 have shown that relatlimab causes no significant improvement in survival or response rate compared to the standard of care [135,159]. Moreover, activity in microsatellite-stable colorectal cancer has also remained limited, setting a precedent for investigation into the improvement of LAG-3-targeted therapies in GI oncology [136].

#### 4.1.4. Future Perspectives

Although LAG-3 blockade has validated the concept of next-generation checkpoint inhibition, several challenges remain. Predictive biomarkers are still poorly defined, and adaptive resistance mechanisms may reduce long-term therapeutic benefit [160]. Ongoing studies are focusing on biomarker-driven patient selection and rational combination strategies to improve outcomes in GI cancers.

### 4.2. TIGIT

#### 4.2.1. Biology and Mechanisms

T-cell immunoreceptor with immunoglobulin and ITIM domain (TIGIT) is an inhibitory receptor expressed on activated T cells, regulatory T cells, and natural killer (NK) cells [161,162]. TIGIT primarily binds CD155 on tumor cells and antigen-presenting cells, leading to suppression of T-cell activation, cytokine production, and NK-cell cytotoxicity [163]. In addition, TIGIT competes with the co-stimulatory receptor CD226 for CD155 binding, further limiting effective antitumor immune responses [164].

#### 4.2.2. TIGIT and T-Cell Exhaustion

TIGIT is closely associated with T-cell exhaustion and is frequently co-expressed with PD-1 on dysfunctional tumor-infiltrating lymphocytes [165]. Persistent TIGIT signaling contributes to impaired cytokine production, reduced proliferation, and weakened antitumor immunity. TIGIT and PD-1 also converge on complementary inhibitory pathways involving suppression of CD226-mediated co-stimulatory signaling, providing the rationale for dual checkpoint blockade strategies [166]. Preclinical studies showed that combined TIGIT and PD-1 inhibition restored CD8+ T-cell activity more effectively than blockade of either pathway alone [167]. Emerging evidence further suggests that TIGIT contributes to metabolic dysfunction in GI tumors, particularly in gastric and colorectal cancer [168,169]. Translating this concept into clinical applications, though, has proven difficult, with anti-TIGIT drug facing large setbacks due to trials like Roche’s later SKYSCRAPER trials and early Gilead/Arcus STAR-221 trial data in lung, gastric, and esophageal cancers lacking added survival or response benefits over standard anti-PD-(L)1 therapy [170,171].

#### 4.2.3. Clinical Development

Several TIGIT-targeting antibodies, including tiragolumab, vibostolimab, and domvanalimab, are currently under clinical investigation. Although early NSCLC studies reported encouraging activity with tiragolumab plus atezolizumab, later phase III trials produced mixed results, highlighting the complexity of TIGIT biology and challenges in patient selection [172,173,174].

More encouraging results have emerged in GI malignancies. The phase III SKYSCRAPER-08 trial demonstrated improved progression-free survival with tiragolumab plus atezolizumab and chemotherapy in unresectable esophageal squamous cell carcinoma [175]. Similarly, the MORPHEUS-EC study reported promising response rates with TIGIT-based combinations in advanced esophageal cancer [176].

#### 4.2.4. GI Oncology Relevance and Future Perspectives

TIGIT expression has been identified in several GI malignancies, including gastric cancer, colorectal cancer, hepatocellular carcinoma, and esophageal cancer, particularly in advanced or treatment-resistant disease [177]. High CD155/TIGIT expression has been associated with poor prognosis and immunosuppressive tumor microenvironments, while frequent co-expression with PD-1, LAG-3, and TIM-3 suggests a broader role in immune exhaustion and adaptive resistance [177,178].

Despite strong biological rationale, clinical results with TIGIT inhibitors remain variable. Ongoing research is focused on identifying predictive biomarkers, optimizing combination strategies, and better understanding tumor-specific TIGIT biology to improve therapeutic benefit in GI cancers [179,180].

### 4.3. TIM-3

#### 4.3.1. Biology and Mechanisms

T-cell immunoglobulin and mucin-domain containing-3 (TIM-3) is an inhibitory immune checkpoint receptor expressed on exhausted T cells, regulatory T cells, natural killer cells, macrophages, and dendritic cells [181,182]. TIM-3 interacts with several ligands, including Galectin-9, HMGB1, phosphatidylserine, and CEACAM1, leading to suppression of T-cell activation, cytokine production, proliferation, and cytotoxic function [183]. Beyond its effects on T cells, TIM-3 also impairs dendritic-cell antigen presentation and contributes to NK-cell dysfunction and myeloid-mediated immune suppression within tumors.

#### 4.3.2. TIM-3 and Adaptive Resistance

TIM-3 is strongly associated with terminal T-cell exhaustion and is frequently co-expressed with PD-1 on dysfunctional tumor-infiltrating lymphocytes [184]. Upregulation of TIM-3 following PD-1 blockade has emerged as an important mechanism of adaptive resistance to immunotherapy. Preclinical studies showed that TIM-3 expression increased during progression on anti-PD-1 therapy, while subsequent TIM-3 blockade partially restored antitumor immune activity [108]. Combined inhibition of TIM-3 and PD-1 demonstrated synergistic restoration of T-cell function and improved tumor control compared with inhibition of either pathway alone [184].

#### 4.3.3. Clinical Development

Several TIM-3-targeting agents, including cobolimab, sabatolimab, and INCAGN02390, are currently under clinical investigation. Early phase studies demonstrated manageable safety profiles and modest antitumor activity, particularly when combined with PD-1 blockade [185,186]. Cobolimab combined with dostarlimab has shown encouraging activity in selected GI malignancies, including advanced hepatocellular carcinoma and biliary tract cancer [187,188]. However, no TIM-3-targeting therapy has yet received regulatory approval.

#### 4.3.4. GI Oncology Relevance and Future Perspectives

TIM-3 overexpression has been associated with poor prognosis and immunosuppressive tumor microenvironments in hepatocellular carcinoma, gastric cancer, colorectal cancer, and biliary tract cancer [189]. In these tumors, elevated TIM-3 expression has been linked to exhausted CD8+ T-cell phenotypes, resistance to therapy, and worse clinical outcomes [130,190,191].

Although TIM-3 remains a promising therapeutic target, its complex signaling network and broad expression across immune cell populations make prediction of therapeutic response challenging [182]. Ongoing research is focused on biomarker-driven patient selection and rational combination strategies involving PD-1 blockade and modulation of the tumor microenvironment to improve outcomes in GI malignancies.

### 4.4. VISTA

#### 4.4.1. Biology and Mechanisms

V-domain Ig suppressor of T-cell activation (VISTA) is an inhibitory immune checkpoint mainly expressed on myeloid cells, including macrophages, myeloid-derived suppressor cells, and dendritic cells [139,192]. Unlike PD-1 and CTLA-4, which primarily regulate T-cell responses, VISTA plays a central role in myeloid-mediated immune suppression within the tumor microenvironment [193]. VISTA signaling suppresses T-cell activation and promotes immunosuppressive macrophage and regulatory T-cell activity, contributing to immune tolerance in tumors.

#### 4.4.2. VISTA and Immune Suppression

High VISTA expression has been associated with “cold” and immune-excluded tumors characterized by poor T-cell infiltration and dominant myeloid suppression [109]. VISTA is frequently co-expressed with other inhibitory checkpoints and appears particularly relevant in pancreatic and hepatobiliary malignancies, where myeloid-driven immune suppression is prominent.

#### 4.4.3. Clinical Development and GI Oncology Relevance

Several VISTA-targeting therapies, including HMBD-002 and SNS-101, are currently in early clinical development and have shown encouraging safety profiles in phase I studies [194,195]. VISTA expression has been identified in pancreatic cancer, gastric cancer, hepatocellular carcinoma, and biliary tract cancers, where it has been associated with suppressive tumor microenvironments and poorer clinical outcomes [196,197]. Preclinical studies further suggest that VISTA blockade may improve CD8+ T-cell infiltration and partially restore antitumor immunity in resistant GI tumors.

#### 4.4.4. Future Perspectives

Although clinical development is still early, VISTA remains a promising target in immune-resistant GI cancers, particularly those driven by strong myeloid-mediated suppression. Future studies will likely focus on combining VISTA inhibition with PD-1 blockade and other tumor microenvironment-targeting strategies.

## 5. Combination Immunotherapy Strategies

The complexity of immune resistance in GI cancers has led to increasing interest in combination immunotherapy strategies, as seen in Table 2, with multiple resistance mechanisms being targeted simultaneously. These approaches aim to restore antitumor immunity, improve immune cell infiltration, and overcome resistance to single-agent checkpoint blockade [117,198].

### 5.1. Dual Checkpoint Blockade

Dual checkpoint inhibition combines PD-1/PD-L1 blockade with inhibition of additional checkpoints such as LAG-3, TIGIT, TIM-3, or CTLA-4. Because exhausted T cells frequently co-express multiple inhibitory receptors, combined blockade may enhance antitumor immune responses more effectively than monotherapy [7,8]. In MSI-H/dMMR metastatic colorectal cancer, nivolumab plus ipilimumab demonstrated substantial clinical benefit [50]. Similarly, the SKYSCRAPER-08 trial reported improved progression-free survival with tiragolumab plus atezolizumab and chemotherapy in esophageal squamous cell carcinoma [175]. However, efficacy remains limited in many immune-resistant tumors, particularly MSS colorectal cancer.

### 5.2. ICIs Combined with Chemotherapy

Chemotherapy may enhance immunotherapy responsiveness by promoting immunogenic cell death and increasing tumor antigen release [63,200]. Oxaliplatin-based regimens appear particularly effective in stimulating immune activation in GI malignancies [201]. Clinically, PD-1 inhibitors combined with chemotherapy are now established first-line treatment options in advanced gastric, gastroesophageal junction, and esophageal cancers following positive studies such as KEYNOTE-859, showing a 1.4-month increase in overall survival (OS), a 9.5% increase in objective response rate (ORR) and a 1.3-month increase in progression-free survival (PFS) [52,202].

### 5.3. ICIs Combined with Targeted Therapy

Targeted therapies may synergize with ICIs through modulation of tumor vasculature and reduction in immunosuppressive signaling. Anti-angiogenic agents are among the most widely studied partners because VEGF inhibition may improve immune cell infiltration and reduce myeloid-mediated suppression [203,204]. In hepatocellular carcinoma, atezolizumab plus bevacizumab significantly improved survival compared with sorafenib and became a first-line standard of care [113,205]. HER2-targeted therapies combined with PD-1 blockade are also under investigation in HER2-positive gastric and gastroesophageal cancers [206]. This has shown the most promise in studies like KEYNOTE-811 showing improved ORR and progression-free survival rates of 75% and 12.1 months, respectively, and current studies like DESTINY-Gastric03 work to investigate this even more within gastric cancer within first-line applications [207,208].

### 5.4. ICIs Combined with Radiation Therapy

Radiation therapy may enhance antitumor immunity through neoantigen release and activation of cGAS-STING signaling pathways [209,210]. In locally advanced rectal cancer, incorporation of ICIs into radiotherapy-based neoadjuvant strategies has shown encouraging pathological response rates in early studies [211]. In metastatic MSS colorectal cancer, radiation may partially sensitize tumors to checkpoint blockade, although overall efficacy remains modest due to RT-induced immunosuppression and fractionation dependence lowering efficiency, while the abscopal effect that leads to destruction of distant metastases sees little clinical translation, with only 10–15% of lung cancer patients receiving PEMBRO + RT experiencing this effect [212,213].

### 5.5. Bispecific Antibodies and Novel Platforms

Bispecific antibodies are designed to target multiple immune pathways within a single therapeutic construct. Cadonilimab, a PD-1/CTLA-4 bispecific antibody, has demonstrated encouraging activity across several GI malignancies, including esophageal, gastric, hepatocellular, and pancreatic cancers [214]. Other emerging approaches include PD-1/LAG-3 bispecific antibodies such as tebotelimab, multispecific antibodies targeting TIGIT and LAG-3, engineered cytokines, and bifunctional fusion proteins [215,216].

Overall, combination immunotherapy represents a promising strategy for overcoming immune resistance in GI oncology. However, future progress will likely depend on biomarker-driven patient selection, rational combination design, and careful toxicity management rather than simply increasing the number of immune-targeting agents used together.

## 6. Biomarkers and Patient Selection

Biomarker development remains essential for improving immunotherapy outcomes in GI oncology, as responses to checkpoint inhibitors vary considerably across tumor types and molecular subgroups. Current clinical practice mainly relies on MSI/dMMR status, PD-L1 expression, and tumor mutational burden (TMB), while newer approaches increasingly incorporate circulating tumor DNA (ctDNA), immune gene signatures, spatial immune profiling, and multi-omics models to improve patient selection [52,142].

### 6.1. MSI-H and dMMR

MSI-H/dMMR status remains the most established predictive biomarker for checkpoint inhibitor responsiveness in GI cancers, particularly colorectal cancer [153,217]. Trials such as KEYNOTE-177 and CheckMate 8HW demonstrated substantial benefit from PD-1 blockade and dual checkpoint inhibition in MSI-H/dMMR metastatic colorectal cancer [50,93]. However, these tumors represent only a small proportion of metastatic GI cancers, and resistance can still develop despite their favorable immunogenic profile [100].

### 6.2. PD-L1 Expression

PD-L1 expression, commonly assessed using the combined positive score (CPS), is widely used in gastroesophageal cancers. In general, higher PD-L1 expression is associated with greater benefit from PD-1/PD-L1 blockade [76,143]. However, PD-L1 remains an imperfect biomarker because of assay variability, spatial and temporal heterogeneity, and dynamic expression changes after treatment or inflammation [144]. Therefore, PD-L1 is best interpreted alongside other clinical and molecular features rather than as a standalone predictor.

### 6.3. Tumor Mutational Burden

TMB reflects the number of somatic mutations within a tumor and may correlate with neoantigen generation and tumor immunogenicity. Although high TMB has been associated with improved response to checkpoint blockade in some cancers, its prognostic and predictive value in GI malignancies remains inconsistent [119,218]. In microsatellite-stable GI tumors, high TMB alone does not reliably predict immunotherapy benefit, highlighting the importance of additional factors such as antigen presentation and immune cell infiltration [24,219].

### 6.4. ctDNA and Emerging Biomarkers

ctDNA is emerging as a promising biomarker for monitoring treatment response, detecting molecular residual disease, and identifying resistance mechanisms in GI cancers [220,221]. Early ctDNA decline after immunotherapy initiation has been associated with improved survival outcomes and may help distinguish true progression from pseudoprogression [222]. Additional emerging biomarkers include immune gene-expression signatures, tumor-infiltrating lymphocytes, tertiary lymphoid structures, microbiome composition, and AI-driven multi-omics models [222,223,224]. Together, these approaches are shifting biomarker development toward more integrated and dynamic models of patient selection.

## 7. Challenges and Current Limitations

Despite the growing promise of next-generation checkpoint inhibitors and combination immunotherapy, several major challenges continue to limit their effectiveness in GI malignancies.

### 7.1. Biological Complexity and Heterogeneity of Resistance

Immune resistance in GI cancers is highly heterogeneous and involves multiple overlapping mechanisms, including T-cell exhaustion, stromal exclusion, myeloid-driven suppression, and compensatory checkpoint upregulation [4,19]. In many cases, blockade of a single pathway is insufficient because resistance mechanisms evolve dynamically during treatment, as seen with TIM-3 upregulation following PD-1 inhibition [108,133].

The immune landscape also differs substantially among GI malignancies. Pancreatic cancer is characterized by dense desmoplastic stroma and profound myeloid suppression [97,118], whereas MSS colorectal cancer commonly demonstrates immune exclusion associated with WNT/β-catenin signaling [225,226]. In contrast, MSI-H tumors display a more inflamed but checkpoint-restrained phenotype [100]. Additional heterogeneity between primary tumors and metastatic sites, particularly within the liver microenvironment, further complicates treatment selection [190,227].

### 7.2. Biomarker Limitations and the Need for Dynamic Assessments

Although MSI/dMMR status, PD-L1 expression, and TMB are clinically useful biomarkers, they provide only a limited snapshot of antitumor immunity [120,142]. Current biomarkers fail to fully capture immune cell composition, spatial organization, and dynamic changes during therapy, limiting their ability to predict response to next-generation combination regimens.

Real-time monitoring tools are also lacking. Emerging biomarkers such as circulating tumor DNA (ctDNA) kinetics may help identify adaptive resistance and distinguish pseudoprogression from true progression, although their clinical use remains limited [220,222].

### 7.3. Toxicity and Tolerability of Combination Regimens

Combination immunotherapy increases both the frequency and severity of immune-related adverse events. Dual checkpoint blockade with nivolumab and ipilimumab, while effective in selected settings, is associated with substantially higher rates of grade 3–4 toxicities compared with monotherapy [50,114]. As newer combinations targeting LAG-3, TIGIT, TIM-3, and VISTA continue to emerge, balancing efficacy with tolerability will remain a major challenge, particularly in frail GI cancer populations.

### 7.4. Translational Gaps and Preclinical Modeling

Current preclinical models only partially reproduce the complexity of the human GI tumor microenvironment. Many syngeneic mouse models are more immunogenic than human tumors and may overestimate responsiveness to checkpoint blockade [228,229]. In addition, tumor implantation site, immune cell composition, and microbiome interactions can significantly influence treatment response [230,231,232,233].

To address these limitations, newer approaches including patient-derived organoids, organoid–immune co-culture systems, and advanced humanized mouse models are being developed to better replicate patient-specific tumor biology and evaluate novel immunotherapeutic strategies [234,235].

### 7.5. Cost, Accessibility, and Global Applicability

Next-generation immunotherapies impose a substantial financial burden on healthcare systems and patients. Combination regimens are associated with higher treatment costs, increased hospitalizations, and greater toxicity-related healthcare utilization compared with monotherapy [236,237,238]. Access to biomarker testing, immunotherapy agents, and specialized toxicity management also remains highly uneven across geographic regions.

Participation of low- and middle-income countries in immunotherapy clinical trials remains limited, raising concerns regarding global inequities in access to precision oncology [239,240]. These challenges highlight the need for more cost-effective strategies, broader international collaboration, and improved global access to immunotherapy and biomarker testing [241,242].

## 8. Future Directions

Overcoming immune resistance in GI malignancies will likely require moving beyond single-agent checkpoint inhibition toward more personalized and biologically driven strategies. Future progress will depend on rational combination therapies, improved biomarker selection, and approaches capable of reshaping the tumor microenvironment rather than targeting a single inhibitory pathway alone.

### 8.1. Rational Combination Strategies and Adaptive Trial Designs

Adaptive platform trials are becoming increasingly important in GI oncology as the number of potential immunotherapy combinations continues to expand. The GEMINI-Gastric platform trial is currently evaluating several combinations simultaneously, including PD-1/CTLA-4, PD-1/TIGIT, and PD-1/TIM-3 strategies combined with chemotherapy [243]. Future studies may also stratify patients according to immune phenotypes such as immune-inflamed, immune-excluded, and immune-desert tumors rather than relying only on histology-based classification [244].

### 8.2. Cellular Therapies and Vaccines

For highly resistant tumors such as pancreatic cancer and MSS colorectal cancer, reinvigorating existing T cells alone may not be sufficient. TCR-engineered T-cell therapies targeting KRAS mutations have shown early activity in pancreatic cancer, while personalized neoantigen vaccines generated durable CD8+ T-cell responses in resected pancreatic tumors [245,246,247,248]. Similar vaccine-based approaches are now being explored in MSS colorectal and gastric cancers.

### 8.3. Tumor Microenvironment and Microbiome Modulation

Targeting stromal and immunosuppressive components of the tumor microenvironment remains a major area of interest in GI oncology. Recent studies suggest that selective targeting of cancer-associated fibroblast populations may improve immune cell infiltration and enhance responsiveness to checkpoint blockade [249].

The gut microbiome has also emerged as an important regulator of immunotherapy response. Certain bacterial taxa have been associated with improved outcomes, while early studies suggest that fecal microbiota transplantation combined with checkpoint blockade may partially restore immunotherapy sensitivity in resistant tumors [250,251,252].

### 8.4. Advanced Biomarkers and Novel Therapeutic Platforms

Future biomarker development will likely move beyond single-marker models toward integrated approaches incorporating spatial transcriptomics, multiplex imaging, ctDNA kinetics, and AI-driven multi-omics profiling [253,254]. In parallel, next-generation therapeutic platforms, including bispecific antibodies, engineered cytokines, and conditionally activated antibodies, are being developed to improve antitumor efficacy while limiting systemic toxicity [243,255].

### 8.5. Epigenetic and Metabolic Reprogramming

Epigenetic remodeling and metabolic targeting are emerging as additional strategies to reverse T-cell exhaustion and improve immunotherapy responsiveness. Early studies suggest that agents such as low-dose decitabine may partially restore exhausted T-cell function, while metabolic interventions targeting lactate metabolism and arginine depletion are also under investigation [64,141,256,257].

### 8.6. ICI Safety Profile Investigation

While ICIs offer durable efficacy, they induce unique immune-related adverse events (irAEs). In GI oncology, common irAEs include immune-mediated colitis, hepatitis, pneumonitis, and endocrinopathies. Dual checkpoint therapies have the potential to increase the rates of these toxicities compared to immune monotherapy, with one study showing markedly increased toxicity rates in dual ICI therapy in over half of the tested patients [258]. Expanded investigation on this topic is required to find if long-term safety profile risks outweigh those in ICI monotherapy [259].

## 9. Conclusions

Immune checkpoint inhibitors have transformed the treatment of a subset of gastrointestinal cancers, most notably MSI-H/dMMR tumors, but the vast majority of GI malignancies remain refractory to current immunotherapies. The profound immune suppression driven by stromal, myeloid, metabolic, and adaptive resistance mechanisms requires a shift from single-agent checkpoint blockade to rational, multi-modal therapeutic strategies. Next-generation inhibitors targeting LAG-3, TIGIT, TIM-3, and VISTA, together with combinations incorporating chemotherapy, anti-angiogenic agents, radiation, and emerging cellular and microbiome-based therapies, represent a promising path toward expanding the benefit of immunotherapy.

However, the future of GI immuno-oncology will depend not only on developing new drugs but on building an integrated framework of dynamic, composite biomarkers, adaptive clinical trial designs, greater analysis of the scientific rationale behind treatments, and deeper immunological and biological understanding of patient management to maximize clinical benefit. Precision immunotherapy, matching the right combination to the right patient at the right time, guided by the immune contexture of the individual tumor, is the goal. With the advances outlined in this review, the field is moving closer to realizing durable immune-mediated disease control across the full spectrum of gastrointestinal malignancies.

## Figures and Tables

**Table 1:** Immune Landscape Features Across Major Gastrointestinal Malignancies. The immune landscape varies substantially across GI malignancies, driven by differences in tumor mutational burden (TMB), microsatellite instability (MSI) prevalence, PD-L1 expression, dominant immune cell populations, and stromal composition. These features directly influence immunotherapy responsiveness and prognosis.

Feature	Esophageal Cancer	Gastric Cancer	Colorectal Cancer	Pancreatic Cancer (PDAC)	Hepatocellular Carcinoma (HCC)	Cholangiocarcinoma (CCA)
**MSI** **-H/dMMR Prevalence**	5% (very rare in ESCC; slightly higher in EAC) [68]	5–20% (highest in intestinal subtype) [69]	10–15% overall; up to ~15% in right-sided colon [68,69]	1–2% (very rare) [70,71]	Very rare (<1%); no established role for MSI testing [69]	2–3% (rare) [69]
**Median TMB**	Moderate; ~57% TMB-H in ESCC (≥10 mut/Mb); MSI-H very rare [72,73]	Moderate; bimodal distribution (MSI-H subset is TMB-high) [72]	Bimodal: MSI-H subset TMB-high (14.6% right colon); MSS subset TMB-low [68]	Low overall; ~1.3% TMB-H; TMB-high subset enriched in long-term survivors [74]	Low to moderate; no established role for TMB testing [75]	Low; limited data
**PD-L1 Expression**	~54% positive (CPS ≥1) in ESCC; TPS strongest predictor in SCC [73,76]	CPS ≥1 in ~40–60%; CPS strongest predictor (after MSI-H) [76]	Higher in MSI-H (20.6%) vs. MSS (7.8%); not routinely used for ICI selection in MSS [68]	Low; PD-L1+ more common in dMMR/MSI-H subset [68]	Not used for patient selection; ICI benefit seen regardless of PD-L1 [75]	Variable; higher in TLS-positive tumors [77]
**Dominant Immune Infiltrate**	Immunosuppressive: enriched mMDSCs in EAC; lower Ki67+ CD8 T cells vs. gastric [78]	MSI-H/EBV+: dense CD8+ T cells, Tregs, NK cells; GS subtype: CD4+ T cells, macrophages, B cells; CIN: CD8+ at invasive margin only [79,80]	MSI-H: Tc1/Th1-rich, high CD8+, dendritic cells [81] MSS: Treg, Th2, Th17 dominant [82]	Myeloid-dominant (~42% myeloid cells); abundant tumor-associated neutrophils; sparse T cells [82]	Heterogeneous; T-cell exhaustion prominent; EGR1+ macrophages enriched; CTNNB1 mutations associated with immune exclusion [83]	Dense CAFs; immunosuppressive B cells in tumor; TLS-positive subset has inflamed phenotype [84,85]
**Key Immune Evasion Mechanisms**	Immune suppression at esophageal location; mMDSC enrichment [6,78]	HLA deficiency (especially MSI); TGF-β/Wnt/β-catenin; VEGF-A upregulation in non-responders [6,86]	MSS: T-cell exclusion, Treg/Th17 dominance; MYC/CCNE1 amplification in immune-cold CIN [6]	Dense desmoplastic stroma; CAFs; Tregs; M2 macrophages; T-cell exclusion [6]	Wnt/β-catenin pathway activation; T-cell exhaustion (PD-1, TIM-3, LAG-3); tolerogenic liver microenvironment [83]	CAF-mediated B cell dysfunction; IL-6/TGF-β driven immunosuppression; immature B cells in tumor [84,85]
**Immune Infiltrate and Spatial Biomarker Predictive and/or Prognostic Value**	IS-high associated with better OS (5 yr OS 77.6% vs. 65.8%); independent prognostic factor [87]	Prognostic in differentiated-type pStage III GC (HR 0.39 for RFS); not prognostic in undifferentiated type [88]	Strongest evidence: internationally validated; independent of TNM; relative contribution 47% vs. 28% for TNM [89]	Limited data; TMB-high MSS subset shows higher T-cell density and prolonged survival [74]	Not established; ML-based immune cell density shows prognostic value (OS 20.9 vs. 15.3 mo) [90]	TLS presence predicts ICI response (ORR 71% vs. 0%) [77]
**ICI Monotherapy ORR**	~20–35% (PD-L1+ ESCC, 2L+) [73]	MSI-H: 57–86% [91]; EBV+: up to 100% [92]; MSS/PD-L1+: ~15–50% [76]	MSI-H: 33–45% (1 L pembro) [93]; MSS: <5% MSI-H: 62–71% (1 L NIVO + IPI); MSS: ≤5% [50]	MSI-H: 18–75% (variable) [94]; MSS: minimal	~15–20% monotherapy; combination ICI improves outcomes [75]	~10–15% monotherapy; TLS+ subset: 71% [77]

**Key patterns across GI malignancies:** MSI-H prevalence drives ICI responsiveness but varies from ~15–20% (gastric/right colon) to <2% (PDAC/HCC) [68,69]. HCC is unique in that ICI combinations are approved without biomarker selection [75]. The Immunoscore is validated only in CRC [88], while TLS predicts ICI response in cholangiocarcinoma (ORR 71% vs. 0%) (55). PDAC and MSS CRC remain “cold” with myeloid-dominant or T-cell-excluded microenvironments, respectively [81,95].

**Table 2:** Summary of Landmark Clinical Trials Evaluating Immune Checkpoint Inhibitor Combination Regimens in GI Cancers [199].

Molecular Target	Agent	Regulatory/Development Status	Major GI Indications
PD-1	Pembrolizumab	FDA Approved	1 L Gastric/GEJ adenocarcinoma, dMMR/MSI-H Solid Tumors like CRC
PD-1	Nivolumab	FDA Approved	1 L Gastric/GEJ adenocarcinoma, 1 L Unresectable Hepatocellular Carcinoma (+Ipilimumab)
PD-1	Tislelizumab	FDA Approved/Global Phase III	1L Gastric/GEJ adenocarcinoma
PD-1	Dostarlimab	FDA Approved	1L dMMR/MSI-H recurrent or advanced solid tumors
PD-L1	Atezolizumab	FDA Approved	1L Unresectable or Metastatic Hepatocellular Carcinoma
PD-L1	Durvalumab	FDA Approved	1 L Advanced Biliary Tract Cancer (+GemCis), 1 L Unresectable Hepatocellular Carcinoma (+Tremelimumab)
CTLA-4	Ipilimumab	FDA Approved	dMMR/MSI-H Metastatic CRC (+Nivolumab), 1 L Unresectable Hepatocellular Carcinoma (+Nivolumab)
CTLA-4	Tremelimumab	FDA Approved	1 L Unresectable Hepatocellular Carcinoma (STRIDE regimen with Durvalumab)
TIGIT	Tiragolumab	Phase II/III (Clinical-Stage)	1 L Esophageal Squamous Cell Carcinoma and Esophagogastric Cancers (+Anti-PD-L1)
TIGIT	Ocinberlimab (BGB-A1217)	Phase II (Clinical-Stage)	Advanced Esophageal and Gastric Cancers (+Tislelizumab)
LAG-3	Relatlimab	Phase II/III (Clinical-Stage)	Gastric/GEJ Cancers and dMMR/MSI-H Gastrointestinal Tumors (+Nivolumab)
PD-1/LAG-3 (Bispecific)	Tebotelimab	Phase I/II (Clinical-Stage)	HER2-positive Gastric/GEJ adenocarcinoma (+Margetuximab ± Chemo)

## Data Availability

The data supporting the findings of this study are available from the corresponding author upon reasonable request.
